# Evaluation of plant-produced *Clostridium perfringens* type D *epsilon* toxoid in a vaccine against enterotoxaemia in sheep

**DOI:** 10.4102/ojvr.v84i1.1271

**Published:** 2017-04-25

**Authors:** Tinyiko Mokoena, Ereck Chakauya, Michael Crampton, Boet Weyers, Malefa Tselanyane, Tsepo Tsekoa, Rachel Chikwamba

**Affiliations:** 1 Biosciences Unit, Council for Scientific and Industrial Research, South Africa; 2 Department of Plant Science, Forestry and Agricultural Biotechnology Institute, University of Pretoria, South Africa; 3 Onderstepoort Biological Products, Onderstepoort, South Africa

## Abstract

Enterotoxaemia (pulpy kidney) is a common bacterial disease of sheep caused by *Clostridium perfringens* type D *epsilon* toxin. It has mortality rates of up to 30% in non-vaccinated animals. Current vaccines from whole cell cultures are expensive to manufacture and can induce local inflammatory responses in sheep. They usually have reduced immunogenicity because of the difficulty of standardising the inactivation step in vaccine manufacturing. In the current study, we evaluated the safety and potency of a recombinant plant-made *epsilon* toxoid protein (r-Etox) as an affordable and safer alternative vaccine for developing countries. Results of injection site reactions, rectal temperature and toxin neutralisation test in single and prime–boost inoculations of mice, guinea pigs and sheep suggest that the product is not toxic to animals and could protect sheep against enterotoxaemia.

## Introduction

Enterotoxaemia (commonly known as pulpy kidney disease) is a disease of economic importance in sheep, with a mortality rate of between 10% and 30% in non-vaccinated lambs. Although it also affects calves and goats, the symptoms are less severe, causing a non-fatal subacute disease (Morris et al. 2012). The disease is precipitated when sheep are fed a rich ration, such as green pastures, or if the diet is changed abruptly. The protein-rich material stimulates the growth of *Clostridium perfringens* type D, an anaerobic bacterium which occurs in the intestinal tract, to multiply rapidly and to produce highly active *epsilon* toxin (Etx) that has been shown to be the major cause of the disease. Etx is produced as an inactive protoxin which is activated through a complex process involving trypsinisation where 13 and 29 amino acid residues are removed at the N-terminus and C-terminus, respectively (Bokori-Brown et al. 2011; Garcia et al. 2013). The toxin causes an efflux of intracellular K^+^ ions resulting in cell apoptosis and sudden death of the animal (Chandran et al. 2010; Fennessey et al. 2012). Farmers usually encounter dead animals in the morning and post-mortem examination shows oedema in many organs including the brain, heart, lungs and kidneys. Because of the rapid progression of the disease, antibiotics are of no use and vaccination is the only solution (Titball 2009). Currently available vaccines in South Africa are formalin-treated alum or oil emulsion of whole cell culture or bacterial filtrates of *C. perfringens* (C. welchii) type D. These vaccines usually have reduced immunogenicity because of the difficulty of standardising the inactivation step (Mathur et al. 2010), and some components of the toxoids induce local inflammatory responses or allergic reactions (Jiang et al. 2014). Moreover, the media used for anaerobic fermentation of the bacteria are not readily available in many developing countries, including South Africa, where it has to be imported, thereby making the manufacturing process expensive.

Plants have proven to be useful vehicles for producing high volume but low-cost biologics for different applications and could provide a solution to potential production challenges for enterotoxaemia. The world’s first regulatory opportunity for a plant-made vaccine for veterinary purposes occurred in early 2006 when a Newcastle disease virus vaccine for chickens demonstrated technical and industrial feasible (Sparrow et al. 2007). This provided a regulatory opportunity for other plant-made vaccines to be approved. Since then, several viral antigens have been expressed in plants, including rabies, Norwalk virus, anthrax, avian reovirus and avian influenza virus (Gunn et al. 2012). The advantages of using plants as expression systems include vaccine antigen production that is safe, potentially more cost-effective and scalable than current methods (Chen & Lai 2013; Rybicki et al. 2012; Šmídková et al. 2010). Moreover, plant tissues may act as adjuvants and increase immunogenicity of antigens in animals or, alternatively, plant materials can be freeze-dried to increase the antigen dose on a per-gram basis (Wang & Coppel 2008). Moreover, a plant-made veterinary product in powder form can potentially be easily stored and transported under limited or no refrigeration without degradation (Gunn et al. 2012).

Despite all these advantages, poor expression levels of some antigens and the complex regulatory problems surrounding plant-produced vaccines remain a barrier to commercialisation. In a recent study, Rybicki et al. (2012) highlighted some of the hurdles in commercialising products in South Africa including lack of financial support, lack of manufacturing infrastructure, and an uncertain regulatory and entrepreneurial environment for commercial development of novel plant-made products (Rybicki et al. 2012). In the current study, we explore plant-based expression to demonstrate the feasibility of producing affordable vaccines for animals in developing countries.

## Materials and methods

To demonstrate expression, we used transient expression by agroinfiltration of *Nicotiana benthamiana* plant leaves. For purposes of this study, the nomenclature *Etx* refers to the *C. perfringens* type D *epsilon* toxin gene, r-Etx is the recombinant *epsilon* protein produced from the plant matrix before activation by trypsinisation and r-Etox refers to the plant-produced recombinant *epsilon* toxin protein after trypsinisation and formalin inactivation.

To get to the stage of evaluating the toxicity and potency of the r-Etox in animals, the experiments were conducted in three sequential steps viz: transient expression of recombinant *C. perfringens epsilon* toxin (r-Etx) in *N. benthamiana* leaves, purification and toxin inactivation.

### Transient expression of *epsilon* toxin open reading frame in *Nicotiana benthamiana*

**FIGURE 1:**
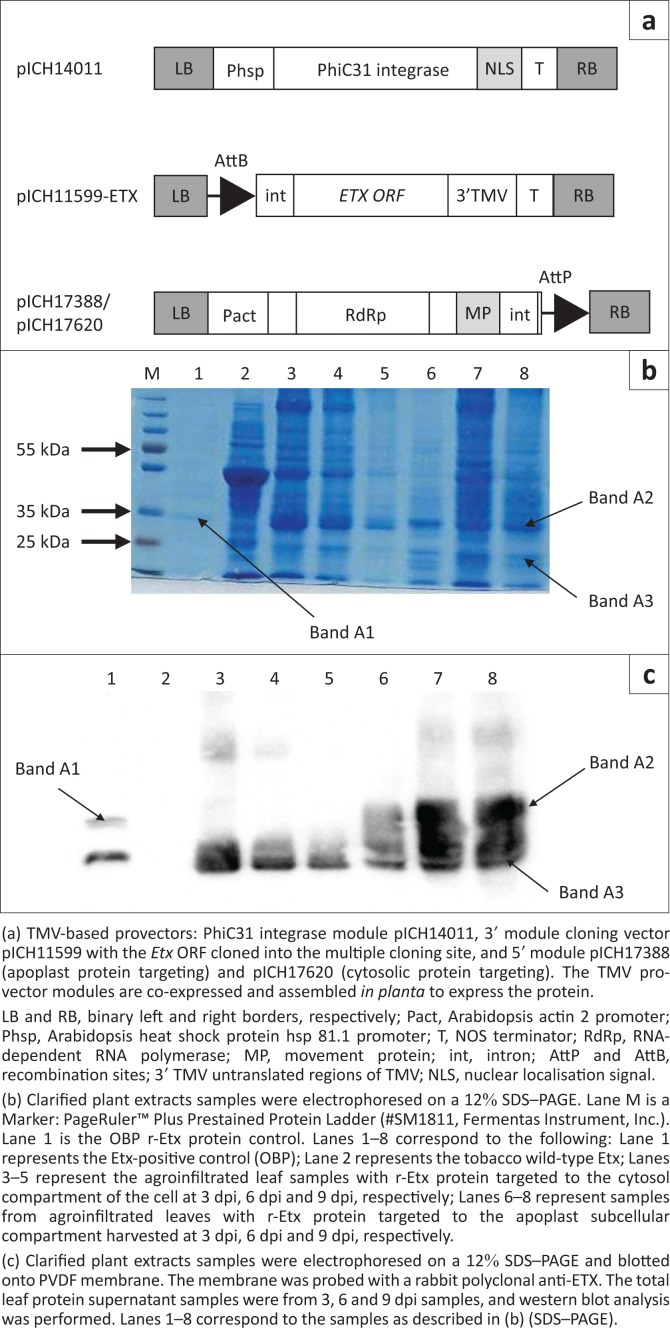
Expression of *epsilon* toxin open reading frame in *Nicotiana benthamiana*. (a) schematic representation of the plasmid constructs, (b) sodium dodecyl sulphate–polyacrylamide gel analysis of the agroinfiltrated recombinant recombinant toxoid and (c) western blot analysis of recombinant toxoid protein.

The r-Etx was produced by expressing a chemically synthesised (GeneArt, GmbH) *N. benthamiana* plant-codon optimised *Etx* open reading frame (ORF) sequence (NCBI: AY858558). The 864-bp sequence is a truncated version of the native gene with 13 N-terminus and 29 C-terminus amino acids removed. The ORF sequences were cloned into the MagnICON vector pICH11599 (Icon Genetics, Halle, Germany) with plCH17388 (apoplast protein targeting) or pICH17620 (cytosolic targeting) together with the plCH14011 (integrase) for transfection into plant leaves (Figure 1a) using *Agrobacterium*-mediated transfer using vacuum infiltration (Giritch et al. 2006). Once *in planta* the integrase assembles the 5′ and 3′ modules into a replication-competent tobacco mosaic virus. The assembled DNA construct is then transcribed and spliced to generate a functional infective replicon that produces the r-Etx protein.

### Protein extraction

Leaf samples (10 g) from transfected *N. benthamiana* plants were harvested between 3, 6 and 9 days post-infection (dpi) and homogenised in extraction buffer (100 mM glycine, 40 mM ascorbic acid, 1 mM EDTA [pH 9.5]) using a 1:1 (w/v) ratio of extraction buffer to harvested plant material (g).

### Protein purification

The resulting green juice was clarified by filtration through four layers of cheese cloth then loaded onto an equilibrated Capto Q column (GE Healthcare, Piscataway, NJ) with three column volumes of buffer A (50 mM diethanolamine, pH 9.5) and the flow-through/wash fraction collected. The unbound proteins were washed and the bound proteins were eluted. The r-Etx-containing fraction was finally ‘polished’ by Superdex 220-pg size-exclusion chromatograph (Bio-Rad, Hercules, CA) in 50 mM diethanolamine buffer, pH 9.5. The r-Etx was then trypsinated and subsequently formalin inactivated as described (Chandran et al. 2010) resulting in a protein code named r-Etox, which was then used for subsequent analysis. Residual toxicity tests and formulation methods were also according to Chandran et al. (2010) with minor modifications. The formalin treatment was conducted to ensure that there was complete processing of the r-Etx into r-Etox as per standard protocols.

### Immunoblot analysis

Purified r-Etx was resolved on 12% reducing sodium dodecyl sulphate–polyacrylamide gel (SDS–PAGE) followed by Coomassie blue staining. The amount of protein loaded was approximately 4 μg – 8 μg of sample. PageRuler™ Plus Prestained Protein Ladder (#SM1811; Fermentas, Hanover, MD) with high-range molecular weight markers was loaded for comparison of the molecular weights. Immunoblot analysis was carried out according to standard protocols where proteins from SDS–PAGE were transferred onto a polyvinylidene difluoride membrane (Bio-Rad, Hercules, CA) then, treated with polyclonal guinea pig anti-Etx antibody (OBP) and rabbit anti-guinea pig IgG-HRP monoclonal antibody (St. Louis, MO). Bound antibodies were detected using ECL detection kit (AEC-Amersham, Midrand, South Africa).

### Enzyme-linked immunosorbent assay analysis

To quantify the accumulation of the r-Etx in the leaves using enzyme-linked immunosorbent assay (ELISA), either six leaf discs from each of three transfected plants or aliquots of the purified samples were used. Leaf discs were ground up in 300 µL of extraction buffer (4 M urea and 100 mM dithiothreitol [DTT]) (Voinnet et al. 2003) in liquid nitrogen and then analysed. ELISA analysis was carried out using crude r-Etx samples and native Etx as protein standards using a protocol derived from Chandran et al. (2010). The r-Etx protein was detected using polyclonal guinea pig Etx antibody (sera) as primary antibody, followed by HRP-conjugated anti-guinea pig polyclonal antibody (Abcam, Cambridge, UK) and detected using calorimetry using an ECL^TM^-plus kit (Amersham Pharmacia, Amersham, UK) according to the manufacturer’s instructions. For comparison, calculated antigen concentrations in the samples were normalised to the fresh leaf weight to obtain the yield of r-Etx per kg fresh weight (FW).

### Protein identification using mass spectrometry

Protein bands of interest were trypsin digested as per the protocol described by Shevchenko et al. (2007). Briefly, gel bands were destained using 50 mM NH_4_HCO_3_/50% MeOH followed by in-gel protein reduction (50 mM DTT in 25 mM NH_4_HCO_3_) and alkylation (55 mM iodoacetamide in 25 mM NH_4_HCO_3_). Proteins were digested overnight at 37 °C using 50 ng – 500 ng tryspin depending on the gel piece size. Digests were resuspended in 35 µL 2% acetonitrile/0.2% formic acid and analysed using a Dionex UltiMate 3000 RSLC system coupled to a QSTAR ELITE mass spectrometer. Peptides were first de-salted on an Acclaim PepMap C18 trap column (75 μm × 2 cm) for 8 min at 5 μL/min using 2% acetonitrile/0.2% formic acid, and then separated on Acclaim PepMap C18 RSLC column (75 μm × 15 cm, 2 µm particle size). Peptide elution was achieved at a flow rate of 500 nL/min with a gradient of 4% – 60% B in 30 min (A: 0.1% formic acid; B: 80% acetonitrile/0.1% formic acid). Nanospray was performed using a MicroIonSpray head assembled with a New Objective, PicoTip emitter. An electrospray voltage of 2.0 kV – 2.8 kV was applied to the emitter. The QSTAR ELITE mass spectrometer was operated in Information Dependent Acquisition using an exit factor of 7.0 and maximum accumulation time of 2.5 seconds. Mass spectrometry (MS) scans were acquired from m/z 400 to 1500 and the three most intense ions were automatically fragmented in Q2 collision cells using nitrogen as the collision gas. Collision energies were chosen automatically as function of m/z and charge.

Protein Pilot v4.0.8085 using Paragon search engine (AB SCIEX, Foster City, CA) was used for comparison of the obtained MS/MS spectra with protein sequences in a Uniswiss 2011 database. Proteins with a threshold above ≥ 99.9% confidence were reported.

### *In vivo* toxicity in mice

The formalin-inactivated protein (r-Etox) was formulated into alum, Montanide gel and ISA 70 (SEPPIC, Paris, France) as 50 µg and 150 µg doses in 0.2 mL volumes. The toxicity of the formulations was evaluated using 5 groups of 10 female CD-1 mice (8–10 weeks old; weighing approximately 37 g each) and eight guinea pigs immunised subcutaneously. Some animals were inoculated with single doses, whereas others in a prime–boost regimen at a 3-week interval between the vaccinations.

### Vaccination and challenge experimental animals

The OBP commercial vaccine (enterotoxaemia alum-precipitated vaccine for sheep and goats) and phosphate-buffered saline were used as the positive and negative controls. All animals were observed for mortalities and bled at weekly intervals until the 35th day. Safety in sheep was determined by subcutaneously injecting groups of two sheep in the inner thigh with 1 mL of formulated candidate vaccines as described above. Subsequently, the animals were observed for an allergic reaction at the injection site and rectal temperatures were measured. We therefore monitored the animals 5 days before and 14 days after vaccination and recorded the size of lesions and the time taken before healing. This was used as an indication of the safety of the vaccine.

The antitoxin units (IU) were determined by mixing the native *epsilon* toxin protein (Etx) with varied dilutions of serum from vaccinated guinea pigs. The residual toxicity in mice was determined by inoculating 0.2 mL of the mixture (dilution range 33 IU/mL – 2.5 IU/mL) intravenously into 10 mice. Lack of mortality in the mice 14 days post-infection indicated neutralisation of Etx. Based on the dilution factor, the number of IU of the test serum was determined and was directly correlated to the potency of the vaccine. A value of 5 IU per mL of serum was considered to be protective according to the European Pharmacopoeia (EDQM 2008).

## Results

To assess expression of the plant-codon optimised *Etx* ORF (Figure 1a), plant extracts from agroinfiltrated plant leaves were analysed by SDS–PAGE (Figure 1b) and western blot analysis (Figure 1c). There is no commercially available pure Ext protein to use as a positive control; hence, a non-formulated vaccine Etx protein (OBP positive control) was used in the analysis. The plant expression results show that the plant-codon optimised *Etx* ORF sequences could be expressed in *N. benthamiana* leaves at levels detectable by SDS–PAGE (Figure 1b). The r-Etx was detectable at a molecular weight of approximately 35 kDa on SDS–PAGE (Figure 1b). This is higher than the predicted 32 kDa for the truncated recombinant protein but migrating at similar molecular weight was the positive control. In the different plant extracts harvested at 3 dpi, 6 dpi and 9 dpi, the band corresponding to the predicted r-Etx (approximately 35 kDa) was clearly visible.

We then used immunoblotting to confirm the identity of the proteins using a polyclonal primary anti-Etx antibody (Figure 1c). A 35-kDa protein was detected in plant extracts at the same molecular weight position as the positive control. In addition, a smaller band corresponding to the 28-kDa molecular weight size band was detectable in the positive control. Surprisingly, a similar band size was detectable in infiltrated plant extracts as detected by both SDS–PAGE and western blot analysis. This suggested a second ORF or possible degradation product.

To further corroborate the results of the SDS–PAGE and western blot analysis, we excised the bands indicated as Band A1 to Band A3 (Figure 1b) and sequenced them by MS. Table 1 shows the sequencing results including the coverage for the three bands. Plant extracts contain complex mixtures of proteins with similar isoelectric points and co-migrating at the same molecular weight on SDS–PAGE. MS can therefore show the predominant proteins in each of the bands. Protein Band A1 (~35 kDa) excised from Lane 1 (see Figure 1b) was confirmed to be predominantly *epsilon* toxin (r-Etx) from *C. perfringens* with 65% protein coverage. The next abundant protein from the band A1 was trypsin, which is used in the peptide digestion during sequencing. The sequencing results confirmed that the protein sample being formulated for the vaccine was predominantly r-Etx. We then sequenced the corresponding protein band in the plant extracts, migrating at the same molecular weight (B and A2), and confirmed that it was also *epsilon* toxin from *C. perfringens* at 68% sequence coverage, followed by two stress-related proteins from the tobacco extract. Band A3 was shown to be glucan-endo-1,3-*beta*-D-glucanase, which is one of the stress-related proteins. In summary, the sequencing results confirmed that the *epsilon* toxin was expressed in plant leaves at levels detectable by SDS–PAGE. The sequencing results also corroborated the SDS–PAGE and western blot analyses.

**TABLE 1:** Sequencing data for the putative r-Etx protein bands from SDS–PAGE by mass spectrometry peptide finger printing.

Band ID	% Coverage	Accession	Name of protein	Species	Peptide (95%)
**A1**	**65**	**S27536**	***Epsilon* toxin**	***Clostridium perfringens***	**25**
**A1**	**26**	**TRPGTR**	**Trypsin (EC 3.2.21.4) precursor**	**Pig**	**5**
**A1**	**8**	**T07140**	**Glucan-endo-1,3 beta-**d**-glucanase (EC 3.2.1.3)**	**Potato**	**2**
A2	68	S27536	*Epsilon* toxin	*Clostridium perfringens*	28
A2	54	D38257	Glucan-endo-1,3 beta-D-glucanase (EC 3.2.1.3)	Common tobacco	10
A2	52	B38257	Glucan-endo-1,3 beta-D-glucanase (EC 3.2.1.3)	Common tobacco	10
A3	55	B34801	Pathogenesis-related protein Q precursor	Common tobacco	14
A3	25	B38J14	Triose phosphate isomerase cytosolic isoform-like	*Solanum tuberosum*	5
A3	21	TRPGTR	Trypsin (EC 3.2.21.4) precursor	Pig	5

Proteins with the highest coverage are represented in bold type.

**FIGURE 2:**
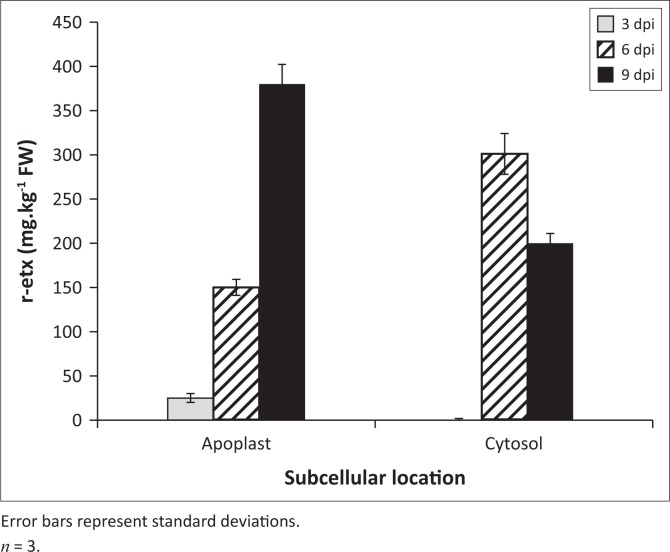
Effect of subcellular targeting of recombinant toxoid protein on accumulation. Transient expression of the recombinant toxoid protein in agroinfiltrated *Nicotiana benthamiana* leaves as measured by an anti-*epsilon* toxin enzyme-linked immunosorbent assay on crude extracts at 3, 6 and 9 days post-infiltration.

Quantification of the protein using ELISA showed that the best protein yield of r-Etx was 380 mg kg^-1^ FW in the apoplast when harvested at 9 dpi. The yield varied with the time of harvesting and subcellular targeting of the protein (Figure 2). With a focus on maximising the protein yield, the apoplast vector combination at 9 dpi was chosen to produce bulk plant material. The protein was purified, trypsinated, inactivated, formulated and injected into animals for safety and potency.

The safety of the candidate vaccines was investigated using a mouse and guinea pig lethality test (Table 2) and also by examining injection site reaction as indicated by the diameter and duration of lesions on the injector site reaction. To assess the allergic reactions to the vaccine or adjuvant, the rectal temperatures of the vaccinated sheep was monitored. At 2 weeks post-inoculation, all mice survived the 50 µg or 150 µg r-Etox doses, while all but one group of guinea pigs had 100% survival (Table 2). None of the inoculated mice and guinea pigs showed clinical signs of disease at the time of observation irrespective of the vaccine formulation. Furthermore, we examined the safety of the candidate vaccines in sheep (Table 3). The rectal body temperature of vaccinated sheep remained within the same range as the positive control. All groups except the 150 µg ISA 70 and the control group had no injection site reaction to the vaccination (Table 4). The lesions in the 150 µg ISA 70 group started at 3 days post-inoculation and lasted until 12 days. The commercial vaccine group (Alum) had a similar pattern with injection site reaction observed 8 days post-vaccination and lasting up to 5 days. Despite these variations, the vaccine’s overall inference was that the vaccine formulations were safe in animals.

**TABLE 2:** Safety of plant-based enterotoxaemia vaccine candidates in mice and guinea pigs.

Adjuvant	Antigen dosage (µg r-Etox)	% Survival	
		Mice	Guinea pigs
Alum	50 µg prime	100	100
	150 µg prime	100	100
	50 µg prime + 50 µg boost	ND	100
	150 µg prime + 150 µg boost	ND	100
ISA 70	50 µg prime	100	100
	150 µg prime	100	100
	50 µg prime + 50 µg boost	ND	100
	150 µg prime + 150 µg boost	ND	100
Montanide	50 µg prime	100	100
	150 µg prime	100	100
	50 µg prime + 50 µg boost	ND	88
	150 µg prime + 150 µg boost	ND	100
Positive control	-	ND	100

Mice and guinea pigs were intravenously injected with 0.2 mL and 2 mL of the vaccine formulation, respectively. The mice (*n* = 50) only received a primary vaccination, whereas the guinea pigs (*n* = 8) were also boosted at 21 days after primary vaccination. The animals were observed over 14 days post-vaccination for mortality.

ND, not determined.

**TABLE 3:** Safety of enterotoxaemia candidate vaccines in sheep.

Adjuvant	Antigen dosage (µg r-Etox)	Rectal temperature (°C)		
		Average	Min	Max
Alum	50	39.2	38.5	40.1
	150	39.3	38.6	40.0
ISA 70	50	39.4	38.6	40.1
	150	39.2	38.2	40.2
Montanide	50	39.2	38.5	401.
	150	39.4	38.5	40.5
Positive control (Alum)	-	39.3	38.5	40.2

Lambs were vaccinated with 2 mL of vaccine subcutaneously in the thighs, and the animals were observed for allergenic reaction as indicated by the rectal temperatures. Rectal temperature was monitored from 5 days pre-vaccination up to 14 days post-vaccination.

**TABLE 4:** Safety of enterotoxaemia candidate vaccines in sheep.

Adjuvant	Antigen dosage (µg r-Etox)	Diameter of lesions			Duration (days)
		Min (cm)	Max (cm)	Earliest onset (day)	
Alum	50	0	0	0	0
	150	0	0	0	0
ISA 70	50	0	0	0	0
	150	3	5	3	12
Montanide	50	0	0	0	0
	150	0	0	0	0
Positive control	-	0	4	8	5

Lambs were vaccinated with 2 mL of vaccine subcutaneously in the thighs, and the animals were observed for injection site reaction due to vaccine (measured a diameter and duration of lesions) monitored from 5 days pre-vaccination up to 14 days post-vaccination.

**FIGURE 3:**
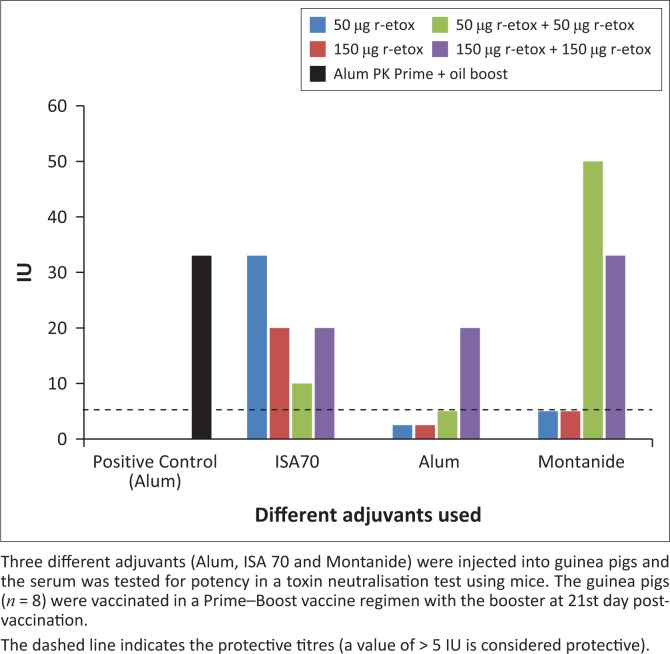
Efficacy of recombinant toxoid vaccine candidates using serum of vaccinated guinea pigs.

We then investigated the potency of the candidate subunit vaccines using the toxin neutralisation assay as per the European Pharmacopoeia and as described by Lobato et al. (2010). The toxin neutralisation assay result was acceptable for regulatory approval of candidate enterotoxaemia vaccines. Results of the toxin neutralisation test showed that vaccination with the r-Etox led to a steady rise in the serum potency to the acceptable level of 5 IU in most regimens (Figure 3). Alum was generally the least potent adjuvant with single vaccinations not reaching the potency level at 3 weeks after vaccination (Figure 3).

The alum formulations did reach the acceptable potency threshold in a prime–boost vaccination regimen. All formulations with the ISA 70 and Montanide adjuvants were potent with values 10 IU – 33 IU. We also observed that the best serum potency was with 50 µg of antigen of Montanide formulation in a booster vaccination. Increasing the amount of antigen to 150 µg did not result in an increase in serum potency (Figure 3). Overall, the results suggest that all the vaccine formulations tested were potent enough to translate to a protection level similar to or better than the commercial vaccines in the field. In all cases, the negative controls showed no serum potency and remained negative throughout the experiment.

## Ethical considerations

All experiments were approved by the necessary ethics committees at the Council of Science and Industrial Research (CSIR) and OBP. As per the advice, if the prime vaccination was safe, no boost vaccine was administered to spare the animals from unnecessary pain. To reduce the number of experimental animals, the guinea pigs used for the safety study were also used in the toxin neutralisation assay.

## Discussion

Native *epsilon* toxin from *C. perfringens* type D is a highly toxic protein that requires enzymatic activation and detoxification by chemical inactivation with formaldehyde as part of vaccine-manufacturing process. This makes it expensive and relatively unsafe to manufacture. Despite considerable efforts in producing a second-generation subunit vaccine (Langroudi, Shamsara & Aghaiypour 2013; Lobato et al. 2010; Souza et al. 2010; Walmsy & Kirky 2008), a plant-based subunit vaccine for enterotoxaemia disease remains unavailable on the market. However, a number of strategies for production of recombinant Etx protein have been reported. The *epsilon* type D toxin has been recombinantly expressed in *Escherichia coli* with the ultimate purpose of producing a vaccine from a relatively less toxic insoluble form of the toxoid (Souza et al. 2010). To date, no reports of production of this important veterinary molecule in plants have been made. In this article, we have successfully expressed the truncated r-Etx in a highly immunogenic form and at potentially viable levels for commercial implementation using a transient deconstructed virus strategy in *N. benthamiana*. Identity of the plant-made r-Etx was confirmed by peptide mass fingerprinting MS. Electrophoresed r-Etx cross-reacted with antibodies raised against the native toxin in a western blot experiment, showing a positive signal at around 35 kDa size (Figure 1b), which is about 3 kDa higher than the predicted mass. This is not very surprising as bands ranging from 26 kDa to 32 kDa have been detected before even with the native *C. perfringens* Etx extracts possibly because of post-transcriptional modifications (Chandran et al. 2010). Our results also show 28 kDa Etx protein band in the positive control as reported in the literature. Interestingly, we detected the 28 kDa in plant extracts by both SDS–PAGE and western blot analysis, but results from sequencing showed undetectable levels of r-Etx. Considering that we used polyclonal antibodies raised in vegetable or grass eating animals, it is conceivable that there is non-specific binding to some plant proteins such as the stress-related glucan-endo-1,3-beta-D-glucanase. However, it is not clear why the plant-expressed r-Ext migrated at a higher molecular weight than predicted. We speculate that the higher molecular weight could be due to glycosylation as reported with other plant-produced proteins (Van Dolleweerd et al. 2014). The r-Etx is predicted to have four *N*-glycoslyation sites at positions 31, 96, 260 and 369. Having said that, the SDS–PAGE, western blot analysis and MS all confirmed the identity of the plant-produced r-Etx ELISA also confirmed the conservation of that the protein to cross-react with Etx-specific antibodies.

To confirm the efficacy and safety of r-Etox, guinea pigs were vaccinated. This study convincingly demonstrated the potency of the plant-made molecule in the animal model using different adjuvants. Interestingly, the use of ISA 70 adjuvant resulted in the most potent formulation where even a single 50-µg dose vaccination led to efficient protection of the animals (Table 2). Increasing the quantity of antigen did not lead to increased potency. Dose titration experiments will be done in future work to properly determine the minimum effective dose for possible commercial implementation. Furthermore, duration of immunity (based on target species serum neutralising antibodies) will be determined during the envisaged field testing.

Based on our results, the potential for commercial production of an enterotoxaemia vaccine in plants is promising. Plant-based expression will present several advantages for production of this subunit vaccine in resource-limited markets. The system has potential for minimising the cost and complexity of production of the bulk antigen for formulation. The typical current production technology for pulpy kidney disease (enterotoxaemia) vaccine relies on *Clostridium* fermentation under anaerobic conditions. The conditions required for optimal production of toxin must be precisely controlled by process engineers (Robertson et al. 2011). In contrast, the technology reported here will use greenhouse cultivation and transfection by vacuum infiltration of *N. benthamiana* plants, processes that require simple invariable conditions and semi-skilled technical staff.

## Conclusion

We have reported time production of enterotoxaemia vaccine based on the *C. perfringens* type D *epsilon* toxoid and empirically demonstrated the safety and antigenicity of the product in animal models from which can be inferred that the product can protect sheep against the disease.
